# Protocol for a systematic review and meta-analysis of the relationship between opioid exposure and dopaminergic measures in humans assessed by nuclear medicine imaging

**DOI:** 10.3389/fpsyt.2026.1800420

**Published:** 2026-04-30

**Authors:** Yulia Shatalov, Tiago Paiva Prudente, Henrique N. P. Oliva, Ahmad Shamabadi, Marc N. Potenza, Gustavo A. Angarita

**Affiliations:** 1 Department of Psychiatry, Yale University School of Medicine, New Haven, CT, United States; 2 Connecticut Mental Health Center, New Haven, CT, United States; 3 Clinical Neuroscience Research Unit, Connecticut Mental Health Center, New Haven, CT, United States; 4 Psychiatric Research Center, Roozbeh Psychiatric Hospital, Tehran University of Medical Sciences, Tehran, Iran; 5 Child Study Center, Yale University School of Medicine, New Haven, CT, United States; 6 Department of Neuroscience, Yale University, New Haven, CT, United States; 7 Connecticut Council on Problem Gambling, Wethersfield, CT, United States; 8 Wu Tsai Institute, Yale University, New Haven, CT, United States

**Keywords:** dopamine, neuroimaging, opioids, positron emission tomography, single photo emission computed tomography, substance use disorder

## Abstract

This protocol outlines planned methodology for a systematic review and meta-analysis of dopaminergic neuroimaging findings in individuals with opioid use disorder (OUD). Converging evidence across substance use disorders (SUDs) shows dopaminergic dysregulation, including low dopamine D_2_ receptor (D_2_R) availability and dopamine release, correlating to anhedonia, altered reward processing, impulsivity, and drug-seeking. Opioid use indirectly alters dopamine signaling, with preclinical work showing dopamine D_2_ receptor and transporter (DAT) changes. However, human Positron Emission Tomography (PET) and Single-Photon Emission Computed Tomography (SPECT) findings are heterogeneous. Therefore, a systematic synthesis is needed to clarify dopaminergic differences related to chronic opioid exposure and their clinical significance. The proposed review will include peer-reviewed studies of adults with diagnosed OUD using PET or SPECT to assess dopaminergic moieties. Databases searched will include PubMed, Scopus, Web of Science, Google Scholar, and Embase. Two independent reviewers will screen records and assess methodological quality. Screening will occur in two stages (title/abstract followed by full-text review) using predefined eligibility criteria. Data will be synthesized descriptively and random-effects meta-analyses will be conducted in Review Manager to estimate pooled dopaminergic measures (e.g., D_2_/D_3_ receptor availability, DAT availability). Statistical heterogeneity will be evaluated using the I_2_ statistic, and sensitivity analyses will assess the impact of methodological variability (i.e. radiotracer type, abstinence duration). This review protocol was registered to the International Prospective Register for Systematic Reviews (PROSPERO; CRD420251229301). Given the substantial global burden of OUD, systematically investigating its neurobiology will provide important mechanistic insights to support the development of novel pharmacotherapies. Although currently approved medications are available, all act on the μ-opioid receptor (μOR) and are associated with moderate treatment retention and frequent relapse, generating the need for additional, mechanistically informed therapeutic targets. This will be the first systematic review and meta-analysis exploring dopaminergic measures in humans with OUD, aiming to offer a clearer understanding of how key dopaminergic markers differ between affected individuals and healthy controls, and explore their potential clinical relevance. Insights generated may inform the development of alternative, non-μOR pharmacological strategies for OUD, including emerging D_3_-selective therapeutics and κ-opioid receptor modulators with the capacity to indirectly influence dopaminergic system function.

## 1 Introduction

The opioid epidemic represents one of the most severe public health crises in the history of the United States, with current estimates indicating that approximately 6.7 to 7.6 million adults in the U.S. are living with opioid use disorder (OUD), and opioid-related overdose deaths have reached unprecedented levels in recent years (1–4). This crisis has been further exacerbated by the widespread infiltration of fentanyl and other synthetic opioids into the illicit drug supply (1, 5). OUD is a chronic, relapsing brain disorder defined by compulsive opioid use that leads to clinically significant impairment or distress (6). Despite decades of clinical and public-health efforts, morbidity and mortality associated with OUD remain high (5).

Currently approved interventions for OUD include methadone, buprenorphine, and naltrexone (7). These act on μ-opioid receptors (μORs) and reduce withdrawal symptoms and/or craving (7). However, treatment retention remains limited, and relapse following discontinuation is common (8–16). Clinical studies report dropout rates greater than 50 percent (17). This highlights the need for novel therapeutic targets addressing the underlying neurobiology of OUD.

The dopaminergic system, implicated in reward processing and motivation, represents one such target of interest (18). Findings across substance use disorders (SUDs) (i.e., alcohol, cocaine, methamphetamines, nicotine, and opioids) show an allostatic weakening of reward systems, characterized by low dopamine D_2_ receptor (D_2_R) availability and low stimulant-induced dopamine release. This literature is both preclinical, using fast scan cyclic voltammetry, fiber photometry, polymerase chain reaction, ribonucleic acid sequencing, western blot, and immunohistochemistry (19–29) and clinical, using Position Emission Tomography (PET) imaging (with [^11^C]-raclopride, [^18^F]-fallypride, or [^11^C]-PHNO) (30–36). Studies link differences in reward-system measures (e.g., decreased D_2_R or increased dopamine transporters (DATs)) to reduced prefrontal glucose utilization, suggesting a mechanism connecting impaired reward signaling with prefrontal circuits critical for salience attribution and inhibitory control (37–39).

In humans with SUDs, [^11^C]-raclopride PET has quantified D_2_R availability and stimulant-induced dopamine release that have been inversely associated with craving sensitivity to rewards, impulsivity, drug self-administration, poor treatment outcomes, and anhedonia (31, 40–45). Additionally, among healthy individuals, greater striatal D_2_R availability is associated with more aversive subjective response to methylphenidate administration, suggesting reduced sensitivity to its reinforcing effects and supporting the interpretation that higher D_2_R availability serves as a protective factor against subsequent addiction risk (46). In depression, blunted striatal dopamine release in response to rewards links to greater severity of motivational anhedonia, implicating impaired dopaminergic signaling in reduced reward sensitivity and pleasure-seeking (47). Collectively, these findings highlight how these PET-derived measures of dopamine system function can help characterize the neurobiological alterations associated with substance use and may guide future research identifying novel pharmacological candidates.

Opioids influence the dopaminergic system by reducing GABAergic inhibition of dopaminergic neurons in the ventral tegmental area, altering dopamine transmission in the nucleus accumbens and other limbic regions (48). This reflects a convergence between opioid, GABAergic, and dopaminergic systems within mesolimbic circuits. Altered dopaminergic signaling in OUD carries important consequences for motivation, reward sensitivity, and behavioral control. Several PET studies using [¹¹C]raclopride have reported reduced striatal dopamine D_2_R availability in individuals with OUD, with differences observed in individuals actively using and those in early abstinence (49, 50). A reduction in dopamine transmission may blunt the experience of natural rewards, producing anhedonia and loss of interest in previously reinforcing activities (50, 51). This diminished reward sensitivity may drive continued opioid use as individuals attempt to restore dopaminergic tone through drug reinforcement.

In addition to the above literature on lower D_2_ receptors, it is important to consider both other dopaminergic alterations and those in other neurobiological systems. For instance, increased activity at D_3_ receptors may inhibit presynaptic dopamine release (52). Thus, addressing alterations in D_3_ receptors represents an emerging therapeutic strategy alongside D_2_ receptors and dopamine transporters (19, 53, 54). In addition, the κ-opioid receptor (κOR) system is closely intertwined with dopamine signaling and the reward deficit model (55, 56). Accordingly, κOR modulators (57, 58) have gained attention for their proposed capacity to indirectly enhance dopamine system function (19, 53, 54, 57, 58). Concurrently, alterations in dynorphin signaling and the κOR system during withdrawal have been proposed to suppress dopamine release and promote dysphoria and negative affect, contributing to an anti-reward state that may increase vulnerability to relapse (57).

Differences in dopamine receptor availability and transporter function in SUDs have also been linked to disadvantageous decision-making and poor self-regulation, including greater impulsivity that may involve preference for smaller, immediate rather than larger, delayed rewards (43, 59–61). These behavioral patterns can contribute to difficulty sustaining abstinence and high relapse risk observed in OUD. Investigating the specific dopaminergic mechanisms underlying these features is therefore important for understanding how chronic opioid exposure may alter brain function and for developing interventions that directly target the neurochemical pathways maintaining OUD.

Preclinical studies provide convergent evidence that chronic opioid exposure disrupts dopamine signaling within broader mesolimbic and cortico-striatal circuits. Prolonged morphine administration in rodents and nonhuman primates reduces D_2_R and D_3_R expression in key reward hubs such as the nucleus accumbens and midbrain, accompanied by attenuated dopamine release and altered firing dynamics of ventral tegmental area (VTA) dopamine neurons (23, 25). Pharmacological manipulation of D_3_R signaling in preclinical studies has been used to explore its role in opioid reinforcement and dopamine regulation. In nonhuman primates, D_3_R partial agonists (e.g., VK4-40) reduces oxycodone self-administration, while in rodents, D_3_R blockade decreases VTA dopamine neuron activity, modulating heroin-evoked dopamine release in the nucleus accumbens (19, 62, 63). Postmortem human studies similarly report altered expression of dopamine receptors, transporters, and dopamine-synthesizing enzymes in individuals with a history of opioid use (64, 65).

In contrast to the extensive literature on dopaminergic differences in people with and without stimulant and alcohol use disorders, where lower dopamine D_2_/D_3_ receptor availability is well established, relatively fewer nuclear neuroimaging studies have examined these measures in OUD, and important questions remain regarding the magnitude, regional specificity, and clinical correlates. Although existing PET and single photon emission computed tomography (SPECT) studies in OUD generally suggest reduced striatal D_2_/D_3_ receptor and DAT availability relative to healthy controls, the evidence is limited by small sample sizes, heterogeneous study designs, and variability in abstinence duration and treatment status (66–68). In addition, the magnitude of these changes remains unclear. This raises the need to systematically examine and integrate the existing neuroimaging evidence to clarify how chronic opioid exposure is related to dopaminergic function in humans. This proposed comprehensive synthesis of PET and SPECT findings can identify consistent patterns and sources of variability across studies, providing a clearer understanding of the extent to which opioids may alter dopamine receptor availability, transporter levels, synthesis, and release. Understanding these relationships is important for linking molecular changes to clinical manifestations such as anhedonia, disadvantageous decision-making, and relapse vulnerability. Ultimately, delineating the dopaminergic mechanisms underlying OUD will support the development of targeted pharmacological and behavioral interventions aimed at altering dopamine system function and improving treatment outcomes for OUD.

To address this gap, the present manuscript outlines a protocol for a systematic review and meta-analysis, establishing a predefined and reproducible framework for quantitively synthesizing PET and SPECT measures of dopaminergic function in individuals with OUD. Given the variability in imaging methodologies and clinical characteristics across studies, specification of eligibility criteria, planned data extraction, and analytical strategies allows for improving comparability and minimizing bias in the resulting synthesis. By formalizing these methodological decisions in advance, this protocol provides a structure for investigating the consistency and clinical relevance of dopaminergic alterations associated with chronic opioid exposure. In doing so, it is intended to facilitate the integration of evidence and support the identification of neurobiological patterns that may inform future research directions and therapeutic development.

## 2 Materials and methods

### 2.1 Research questions

The purpose of this systematic review will be to answer the following research questions: 1) “What dopaminergic differences are observed in individuals with OUD compared to healthy or non-opioid-using controls as measured by nuclear neuroimaging?”; 2) “What are the clinical outcome measures related to these differences in PET or SPECT measures?”; 3) “To what extent do the various reported dopaminergic measures converge on a shared putative neurobiological characteristic, such as a dopamine deficit, and how consistent is this pattern across neuroimaging modalities, ligands, and clinical subgroups?”

### 2.2 Aims and objectives

The aim of this systematic review will be to identify and synthesize the existing evidence on dopaminergic system measures associated with OUD as assessed by PET and SPECT.

The specific objectives of the review will be to:

- Summarize neuroimaging findings on dopamine receptor (D_2_/D_3_R), transporter (DAT), and synthesis/release measures in individuals with OUD.
- Identify correlations between PET or SPECT measures and clinical outcome measures to determine the clinical significance of these dopaminergic measures.
- Compare dopaminergic measures between OUD and healthy control groups to identify consistent neurochemical patterns.
- Evaluate how factors such as opioid type, duration of use, abstinence, or treatment (e.g., methadone, buprenorphine) relate to dopaminergic outcomes.
- Identify methodological strengths, limitations, and gaps in the current neuroimaging literature on OUD to inform future research.

### 2.3 Eligibility criteria

The Preferred Reporting Items for Systematic Reviews and Meta-Analyses Protocols (PRISMA-P) 2015 checklist (69) was used to write this protocol. It can be found in **Supplementary Table 2**. The protocol was registered with the International Prospective Register of Systematic Reviews (PROSPERO) on December 3, 2025. The registration number is CRD420251229301. The protocol contains information such as eligibility criteria, selected databases, data collection, and the risk of bias assessment. For the full systematic review, the PRISMA 2020 checklist (70) will be used when writing to assess a clear and complete report.

#### 2.3.1 Inclusion criteria

Included studies must fit the following criteria:

1) Studies involving human participants aged ≥18 years.
2) Studies including either:
a) participants with a diagnosis of opioid use disorder (OUD) or opioid dependence based on standardized diagnostic criteria, or
b) opioid-naive participants who receive acute opioid administration in a controlled experimental setting.
3) Studies including a comparator group, such as healthy opioid-naïve control participants, placebo conditions, or within-subject comparisons (e.g., pre- vs. post-opioid administration).
4) Studies reporting *in vivo* neuroimaging measures obtained using PET or SPECT assessing dopaminergic function, including:
a) D_2_/D_3_R availability
b) DAT availability
c) Dopamine synthesis capacity (e.g., presynaptic dopaminergic production)
d) Dopamine release (e.g., pharmacological challenge studies)
e) Vesicular monoamine transporter 2 (VMAT2) availability

#### 2.3.2 Exclusion criteria

The following studies will be excluded:

1) Studies based exclusively on post-mortem tissue, autoradiography, or *in vitro* imaging methods.
2) Studies including participants with a primary non-opioid SUD (e.g., involving alcohol, stimulants, benzodiazepines) when that disorder is the primary diagnosis or when its effects cannot be distinguished from opioid use.
3) Studies including participants with neurological or medical conditions known to alter dopaminergic function, such as Parkinson’s disease, Huntington’s disease, schizophrenia, or other neurodegenerative disorders.
4) Studies evaluating agents that do not act as opioids or have negligible opioid activity.
5) Studies lacking a healthy, opioid-naïve control group or another appropriate comparator condition (e.g. placebo) unless data allow for extraction of an internal matched comparison.
6) Studies using neuroimaging methods outside of nuclear medicine (e.g., fMRI, MRS, CT, or structural MRI) or radiotracers that do not measure dopamine receptor binding, DAT availability, dopamine synthesis, or dopamine release.
7) Non-original research, including editorials, commentaries, reviews, single case reports, study protocols without participant data, and conference abstracts without extractable or author-provided data.

### 2.4 Databases to be searched

The electronic databases that will be searched are PubMed, Scopus, Web of Science, Google Scholar, and Embase (Elsevier). Reference lists of included studies will be screened for additional eligible publications.

### 2.5 Search strategy

A comprehensive search strategy was developed and tested on PubMed in October 2025. No restriction will be placed on language or publication year. Articles published in languages other than English will be translated if needed. Any amendments to the search strategy or inclusion/exclusion criteria during the review process will be transparently documented in the final manuscript. A draft PubMed search strategy is included in **Supplementary Table 1**.

### 2.6 Study records

#### 2.6.1 Data management

The files from each database will be imported by the first author into Rayyan, a systematic review management platform, where duplicates will be removed.

### 2.7 Study selection

Two independent review authors (from AS, HNPO, TPP, YS) will select records in two phases. In Phase 1, they will screen titles and abstracts according to the eligibility criteria. In Phase 2, the same reviewers will independently assess the full texts of potentially eligible studies to determine final inclusion.

Discrepancies at any stage will be resolved through discussion. If agreement cannot be reached, a third reviewer (GAA) will be consulted to make the final decision. Moreover, if data are missing or unclear, attempts will be made to contact the study’s corresponding author for clarification. All studies excluded at the full-text stage, along with the reasons for exclusion, will be documented and presented in an appendix in the final review.

### 2.8 Data collection and extraction

Data will be extracted independently by two reviewers (HNPO, YS) using a standardized data extraction form developed in Microsoft Excel. Discrepancies will first be resolved through discussion, and a third reviewer (GAA) will be consulted if consensus cannot be reached. The collected information will include the author(s), year of publication, country, study design, sample size, participant demographics (e.g., age, sex), brain region of interest, opioid use patterns (e.g., time since last use, time since OUD diagnosis, route of drug administration, type of drug used, and others), dopamine outcome measurement (e.g., D_2_R/D_3_R availability, DAT availability), type of nuclear imaging machine, and radiotracer used. Lastly, study limitations, whether reported by authors or identified during the review process, will be recorded.

### 2.9 Risk of bias assessment

The Risk Of Bias In Non-randomized Studies of Exposures (ROBINS-E) tool will be used to assess study quality (71). Moreover, the risk of bias will be assessed independently by two reviewers (from AS, HNPO, YS). Any discrepancies will be resolved through discussion, and if consensus cannot be reached, a third reviewer (GAA) will be consulted.

If required information is missing or unclear, attempts will be made to contact the study authors for clarification. Moreover, risk of bias assessments will be applied at the study level rather than the outcome level, and no study will be excluded solely due to its risk of bias. Summary figures will be created using the *robvis* visualization tool (72).

### 2.10 Data synthesis

A narrative synthesis of the included studies will be conducted to summarize study characteristics, population differences, substance type, and key findings related to dopamine parameters. Methodological variations, including scanning procedures and participant compliance, will be described to contextualize differences across studies.

Where sufficient comparable data are available, meta-analyses will be conducted separately for distinct dopaminergic outcomes (e.g., D_2_/D_3_ receptor availability, dopamine transporter availability, dopamine synthesis capacity, or dopamine release) and brain regions. Outcomes will be grouped according to the biological parameter measured and the brain region in which it was measured. In instances in which the same dopaminergic target is measured using different PET/SPECT outcome units, standardized mean differences will be calculated within these comparable groupings to enable appropriate pooling. Studies measuring fundamentally different biological processes will not be pooled within the same meta-analysis. These will be conducted using random-effects models to account for variability between studies. Random-effects models will be implemented using Review Manager (RevMan), and pooled estimates will be calculated for each dopamine outcome. Effect sizes will be calculated as standardized mean differences with 95% confidence intervals. Statistical heterogeneity will be assessed using the I^2^ statistic. Subgroup analyses and sensitivity analyses will be used to explore potential sources of variability. In addition, sensitivity analyses will be performed to evaluate the robustness of findings. All analyses will be conducted using appropriate statistical software (e.g., SPSS or R).

### 2.11 Analysis of subgroups

The results will be analyzed by subgroups if data are available to explore for variations in PET and SPECT outcomes across specific groups. Planned analyses include population characteristics (e.g., sex), type of dopaminergic measure, and stage of use (e.g., current use or abstinence).

### 2.12 Assessment of certainty evidence

The certainty of evidence across included studies will be evaluated using the Grading of Recommendations Assessment, Development, and Evaluation (GRADE) approach (73). GRADE assesses the overall quality of evidence for each outcome by examining five domains such as risk of bias, inconsistency, indirectness, imprecision, and publication bias. Based on this evaluation, the certainty of evidence for each outcome will be rated as high, moderate, low, or very low. This assessment will inform the interpretation of findings and the strength of conclusions drawn from the review.

## 3 Discussion

The current literature exploring dopaminergic measures in OUD lacks consensus regarding the magnitude and direction of findings. By synthesizing evidence from nuclear neuroimaging studies, this review will provide a rigorous assessment of how key dopaminergic measures, including receptor availability, dopamine synthesis, transporter expression, and dopamine release, may differ in individuals with and without OUD. Beyond characterizing such neurobiological differences, this review will also aim to clarify the clinical relevance of these PET and SPECT findings. By examining the relationship between dopaminergic measures, clinical outcomes, treatment response, and relapse, this work will contextualize imaging findings within the clinical trajectory of OUD.

Importantly, the dopaminergic outcomes investigated in this analysis align with emerging National Institute of Health (NIH) strategic initiatives, such as the “10 Most Wanted” program, which prioritize development of medications targeting the dopaminergic system either directly or indirectly through mechanisms including dopamine D_3_ receptors and the κOR. As such, the insights generated in this review may guide alternative interventional strategies and contribute to a growing momentum toward pharmacotherapies that move beyond the μOR as their primary target.

### 3.1 Implications for research and practice

This review will employ a comprehensive search strategy across multiple databases, use a validated tool for risk of bias assessment, and follow the PRISMA-P 2015 guidelines to ensure transparency and methodological rigor. The insights generated from this review may guide alternative, non-μOR, pharmacological treatment strategies for OUD. Moreover, while no language restrictions will be applied, the availability of full texts or translations may affect inclusion.

### 3.2 Timeline and status of the review

As of this submission, we have piloted the screening process to ensure feasibility of our procedures, but full systematic screening and data extraction have not yet been completed. We anticipate initiating formal title and abstract screening in February with full-text review completed by March 2026. Data extraction and quality assessment are planned for early March 2026. We aim to complete data synthesis and draft initial results by mid-March, with a final report ready by the end of March 2026.

### 3.3 Amendments to the study protocol

There are no planned amendments to this systematic review protocol at the time of writing. However, if any changes become necessary during the review process, they will be documented. Each amendment will include the date of the change, a description of the revision, and the rationale behind it. All amendments will be reflected in both the PROSPERO registration (if applicable) and the final published review to ensure transparency.

## 4 Ethics and dissemination

### 4.1 Ethics

Ethical approval was not required as individual patient data were not collected.

### 4.2 Dissemination

The systematic review will be submitted for publication in a peer-reviewed journal and presented to clinical and research audiences involved in treatment of OUDs. Findings will also be shared through academic conferences, webinars, and collaborative research networks to maximize impact.
